# Exogenous carbon monoxide promotes GPX4-dependent ferroptosis through ROS/GSK3β axis in non-small cell lung cancer

**DOI:** 10.1038/s41420-023-01743-0

**Published:** 2024-01-23

**Authors:** Wei Cao, Mingyu Sun, K. N. Yu, Lele Zhao, Yue Feng, Chunhua Tan, Miaomiao Yang, Ying Wang, Fengqin Zhu, Lianjun Chen, Lili Nie, Ye Zhao, Guodong Chen, Wei Han

**Affiliations:** 1 Anhui Province Key Laboratory of Medical Physics and Technology, Institute of Health and Medical Technology, Hefei Institutes of Physical Science, Chinese Academy of Sciences, 230031 Hefei, P. R. China; 2 Teaching and Research Section of Nuclear Medicine, School of Basic Medical Sciences, Anhui Medical University, 230031 Hefei, P.R. China; 3 Hefei Cancer Hospital, Chinese Academy of Sciences, 230031 Hefei, P. R. China; 4 Department of Physics, City University of Hong Kong, 999077 Hong Kong, P. R. China; 5 State Key Laboratory in Marine Pollution, City University of Hong Kong, 999077 Hong Kong, P. R. China; 6 School of Biology, Food and Environment, Hefei University, 230031 Hefei, P. R. China; 7 Collaborative Innovation Center of Radiation Medicine of Jiangsu Higher Education Institutions and School for Radiological and Interdisciplinary Sciences (RAD-X), Soochow University, 215006 Suzhou, P. R. China

**Keywords:** Non-small-cell lung cancer, Cell death

## Abstract

The gas therapy is drawing increasing attention in the treatment of many diseases including cancer. As one of gas signaling molecules, carbon monoxide (CO) has been proved to exert anti-cancer effects *via* triggering multiple cell death types, such as autophagy, apoptosis and necrosis. Here, we showed that low concentration CO delivered from CO-releasing molecule 3 (CORM-3) effectively induced ferroptosis, known as a novel proinflammatory programmed cell death, in vitro and in vivo. Mechanistically, we found that CO triggered ferroptosis by modulating the ROS/GSK3β/GPX4 signaling pathway, resulting in the accumulation of lipid hydroperoxides and the occurrence of ferroptosis. We think our findings provide novel insights into the anti-cancer mechanisms of CO, and suggest that CO could potentially be exploited as a novel ferroptosis inducer for cancer treatment in the future.

## Introduction

Non-small cell lung cancer (NSCLC) accounts for ~80% of patients of lung cancer, which is the leading cause (~25%) of cancer-related deaths worldwide [1]. Besides traditional treatments for NSCLC including surgery, chemo- and radiotherapy, new treatments, such as targeted therapy and immunotherapy, have been applied in recent years. However, the 5-year survival rate of NSCLC patients is still less than 15%. Consequently, there is an urgent need for the development of novel therapies for treatments of NSCLC.

Recently, the treatment based on gaseous signaling molecules, which are involved in multiple critical functions *via* regulating signal transduction, has got intensive attention. Compared with traditional chemotherapy drugs, gas molecules, such as carbon monoxide (CO), nitric oxide (NO), hydrogen sulfide (H_2_S) and hydrogen (H_2_), display the in-comparable diffusion ability and stronger permeability in cancer therapy [2–4].

CO was always considered as a kind of dangerous and lethal gas due to its strong binding with hemoglobin, then resulting in severe hypoxia [5]. In the past decades, CO has been discovered to be generated by heme oxygenase (HO)-catalyzed heme degradation under physiological conditions. Moreover, it is worth noting that no distinct toxicity was detected after chronic exposure to even 500 ppm CO continuously for up to 2 years in rodents [6, 7]. This also provides the possibility for clinical applications of exogenous low concentration CO. In recent years, low concentrations of endogenous CO have been proved to act as an important physiological signaling molecule to regulate the diverse physiological functions including the regulation of neurotransmitters and neuropeptide release, the relaxation of pulmonary vasculature, the anti-inflammatory effect, etc. In addition, increasing evidences support the anti-cancer effect of exogenous low concentration CO [8–11]. Moreover, CO has been reported to increase the tumor sensitivity to chemotherapy and to provide protection against the doxorubicin-induced cardiotoxicity. It raises more and more concerns that the biological functions and potential application in pharmacology of low concentration CO.

Ferroptosis, an iron-dependent form of programmed cell death (PCD), is characterized by accumulation of lipid peroxides and imbalance of the redox system [12, 13]. Morphologically, ferroptotic cells display mitochondrial atrophy, increased mitochondrial membrane density and reduced mitochondrial cristae [14]. Some ferroptosis-inducing agents or stimulators, including sorafenib, artesunate and piperlongumine, are capable of effectively killing tumor cells, especially highly aggressive tumor cells [15–17]. Furthermore, given the non-apoptotic nature, ferroptosis-based cancer therapy may represent a novel strategy to overcome the resistance to apoptosis-inducing chemotherapy drugs [18, 19]. In addition, as a form of immunogenic cell death, ferroptosis may elicit antitumor immune response *via* releasing various damage-associated molecular patterns [20]. Therefore, inducing ferroptosis has been considered as a promising cancer treatment strategy. Accumulating evidences suggest that GPX4 can neutralize lipid peroxides, thereby protecting cells from ferroptosis. Inhibition or loss of GPX4 triggers ferroptosis by promoting accumulation of lipid peroxides [21]. To date, applications of several GPX4 inhibitors are under different stages of clinical trials [22].

Previous studies have shown that CO could induce autophagy [23], apoptosis [24] and necrosis [25]. In this study, we found that ferroptosis was induced by CO in a time- and dose-dependent manner in NSCLC cells. Mechanistically, we discovered that CO decreased the expression of GPX4 *via* the ROS/GSK3β axis, and then caused the accumulation of lipid peroxides to trigger ferroptosis. Our findings provide novel insights into the antitumor effects of low concentration CO, and demonstrate that the CO might work as a promising therapeutic medical gas against NSCLC cells *via* inducing ferroptosis.

## Results

### CO (CORM-3) induces cytotoxicity in various lung cancer cells

**Fig. 1:**
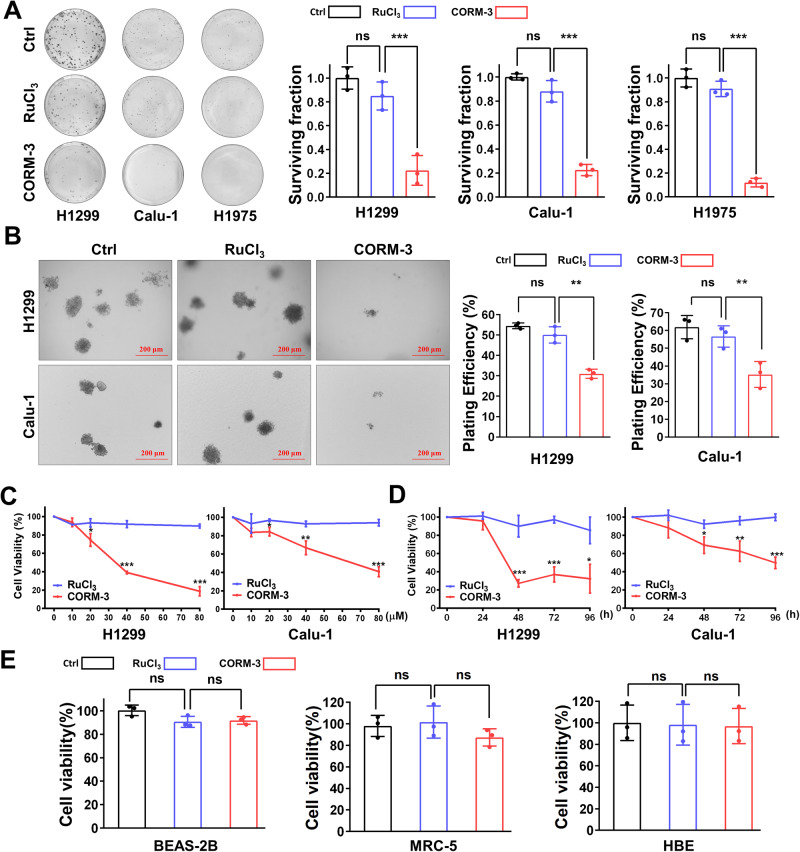
CO (CORM-3) treatment decreased clone survival and cell viability of NSCLC cells. **A** Clone survival and corresponding quantified results of various NSCLC cells treated with CORM-3 (40 μM). **B** Representative images and corresponding quantified results of soft agar clone survival captured at 14 d after CORM-3 treatment (40 μM). **C** Cell viability of H1299 or Ca-lu-1 cells at 96 h after CORM-3 treatment (0–80 μM). **D** Cell viability of H1299 and Calu-1 cells at indicated time points after CORM-3 treatment (80 μM). **E** Cell viability of normal lung cells (BEAS-2B, MRC-5 and HBE) at 96 h after CORM-3 treatment (80 μM). ns not significant, *P < 0.05, **P < 0.01, ***P < 0.001.

To investigate the toxicity of CO to cancer cells, we evaluated the clone formation of various NSCLC (non-small cell lung cancer) cells after CO (CORM-3) treatment. As shown in Fig. 1A, CO treatment significantly decreased the clone survival of H1299, Calu-1 and H1975 cells. Furthermore, clone survival of H1299 and Calu-1 cells in soft agar was also significantly reduced after CO treatment (Fig. 1B).

Next, we evaluated the dose and time dependence of CO-induced cytotoxicity in H1299 and Calu-1 cells with CCK-8 assay. The cell viability was gradually decreased with the increase of CORM-3 concentration (10–80 μM). In addition, the cell viability was also reduced with the prolonged incubation time after CORM-3 exposure (Fig. 1C), and significant cytotoxicity appeared at 48 h after CORM-3 treatment (Fig. 1D). These results suggest that CO significantly kills NSCLC cells in a dose- and time-dependent manner. Importantly, CORM-3 treatment did not affect the cell viability of normal human lung cells (BEAS-2B, MRC-5 and HBE), showing selective cytotoxic effects of CO in cancer cells (Fig. 1E).

### CO (CORM-3) induces ferroptosis in NSCLC cells

**Fig. 2:**
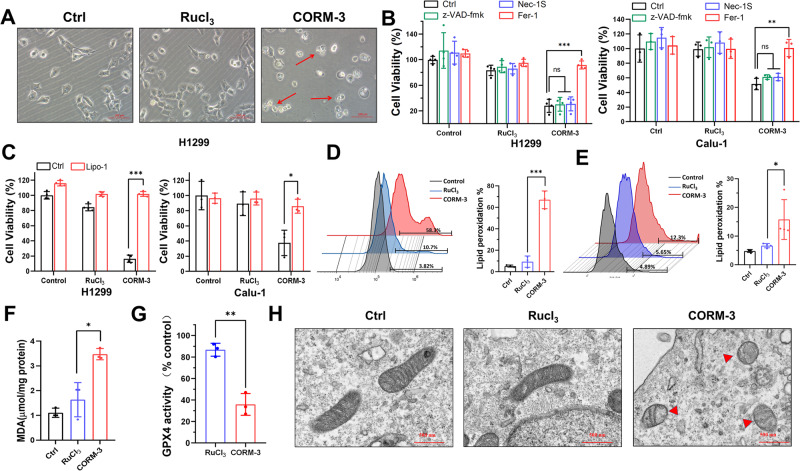
CO induces ferroptosis in NSCLC cells. **A** Representative images of H1299 cells captured at 96 h after treatment with CORM-3 (80 μM). Scale bar: 200 μm. **B** Effect of indicated inhibitors, z-VAD-fmk (20 μM), Nec-1S (20 μM) or Fer-1 (20 μM), on cell viability after CORM-3 (80 μM) treatment in H1299 and Calu-1 cells. **C** Effect of Lipro-1 (10 μM) on cell viability after CORM-3 (80 μM) treatment in H1299 and Calu-1 cells. **D**, **E** Flow cytometry analysis and quantification of cellular lipid peroxidation in H1299 (**D**) and Calu-1 (**E**) cells after CORM-3 (80 μM) treatment. **F** The level of MDA after CORM-3 (80 μM) treatment in H1299 cells. **G** Activity of GPX4 in H1299 cells treated with RuCl_3_ or CORM-3. **H** Mitochondrial morphology captured with transmission electron microscopy after CORM-3 (80 μM) treatment in H1299 cells. ns: not significant, *P < 0.05, **P < 0.01, ***P < 0.001.

After CORM-3 treatment, we observed probable non-apoptotic cell death with microscopy (Fig. 2A). To further explore the type of cell death induced by CO exposure, we treated H1299 or Calu-1 cells with CORM-3 in the absence or presence of different cell death inhibitors. Interestingly, Ferrostatin-1 (Fer-1), a specific ferroptosis inhibitor, nearly completely rescued CO-induced cell death (Fig. 2B). Conversely, apoptosis inhibitor (Z-VAD-fmk) or necroptosis inhibitor (Necrostatin-1s) did not suppress cell death induced by CO. These findings suggest that CO might trigger ferroptosis in NSCLC cells, and this was also supported by the results that liproxstatin-1, another strong lipid oxidation inhibitor, nearly completely attenuated CO-induced cell death in H1299 and Calu-1 cells (Fig. 2C). Meanwhile, the cells treated with CORM-3 also displayed the characteristics of ferroptosis, accumulated lipid peroxidation and elevated MDA level (Fig. 2D–F). In addition, decreased GPX4 activities were observed in H1299 cells at 72 h after CO treatment (Fig. 2G). Moreover, the images of transmission electron microscopy showed morphological features of ferroptosis, the shrunken mitochondria with disrupted cristae in CORM-3-treated cells (Fig. 2H). Collectively, our data suggest that low concentration CO induces ferroptosis in NSCLC cells.

### CO drives ferroptosis through GPX4 downregulation and ROS production

**Fig. 3:**
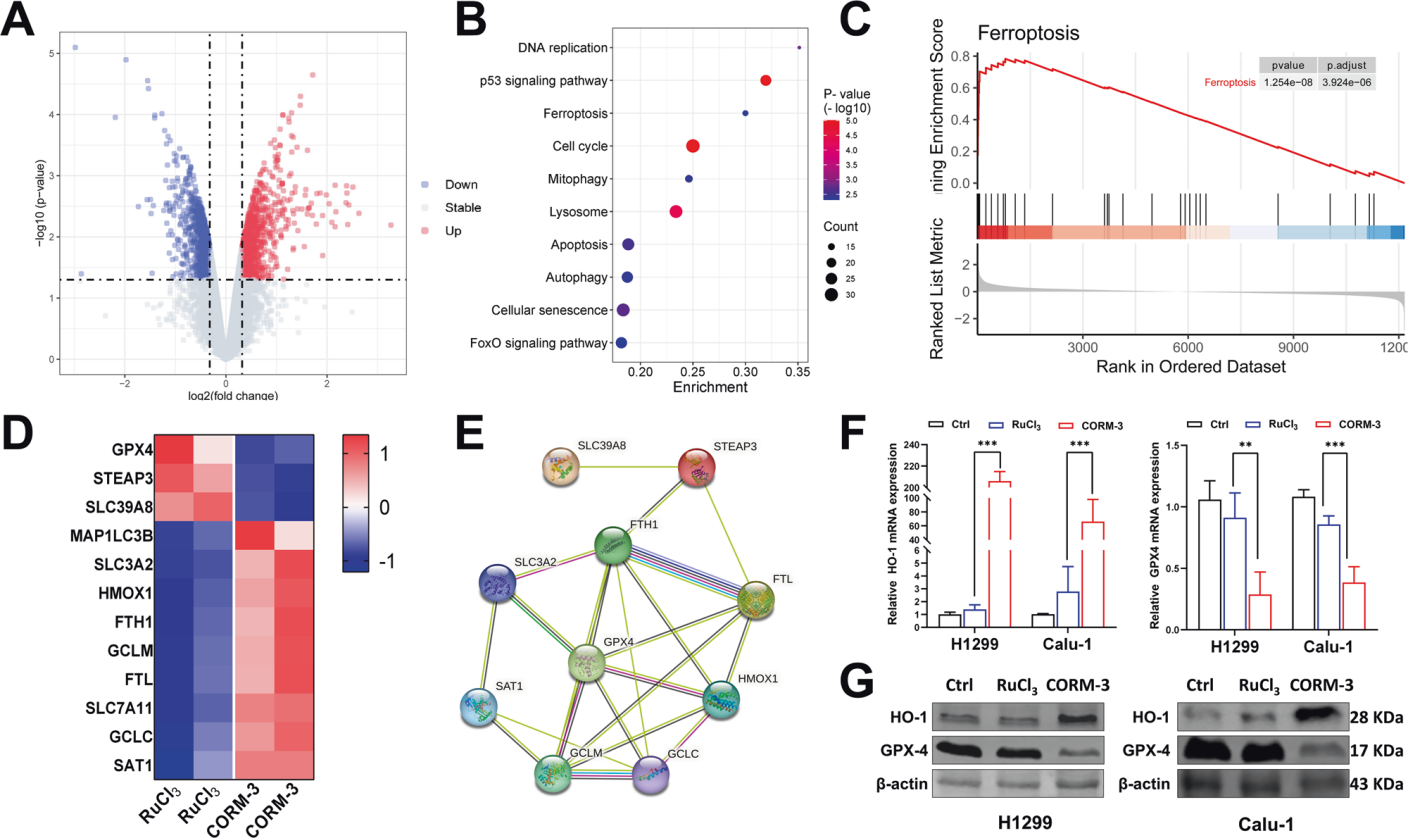
Data analysis of mRNA sequencing of CO-treated H1299 cells. **A** Volcano maps of the differentially expressed genes in H1299 cells (RuCl3 vs CORM-3). **B**, **C** KEGG pathway enrichment analysis (**B**) and GSEA analysis (**C**) were based on the differentially expressed genes in H1299 cells (RuCl_3_ vs CORM-3). **D** Heatmap of ferroptosis-related differentially expressed genes identified by mRNA-seq in H1299 cells (RuCl_3_ vs CORM-3). **E** PPI networks and the significant gene module in the ferroptosis-related gene set. **F**, **G** The mRNA (**F**) and protein (**G**) expression of HO-1 and GPX4 genes after CORM-3 treatment in H1299 and Calu-1 cells. **P < 0.01, ***P < 0.001.

To determine the genes involved in CO-induced ferroptosis, mRNA-seq was performed after RuCl_3_ or CORM-3 treatment in H1299 cells. After preprocessing the original data, with |log2FC | >0.32 and *P* < 0.05 as the standards, 2285 differentially expressed genes (DEGs) were obtained from the data set, including 1078 upregulated genes and 1207 down-regulated genes (Fig. 3A). The results of Kyoto Encyclopedia of Genes and Genomes (KEGG) pathway analysis showed significant enrichment in the ferroptosis pathway after CO treatment (Fig. 3B). Furthermore, Gene Set Enrichment Analysis (GSEA) of these DEGs revealed that ferroptosis signaling pathway was significantly enriched after CO exposure (Fig. 3C). In the ferroptosis pathway, 12 genes including 9 upregulated genes (MAP1LC3B, SLC3A2, HMOX1, FTH1, GCLM, FTL, SLC7A11, GCLC and SAT1) and 3 down-regulated genes (GPX4, STEAP3, SLC39A8) significantly changed in response to CO treatment (Fig. 3D). The PPI network of 12 ferroptosis-related genes was constructed by using the STRING database and the two hub genes, GPX4 and HO-1, were selected through the degree plugin of the Cytoscape software (Fig. 3E). Further validation using qPCR and western blot also confirmed the decreased GPX4 expression and increased HO-1 expression at mRNA and protein levels, in CORM-3-treated H1299 and Calu-1 cells (Fig. 3F, G).

**Fig. 4:**
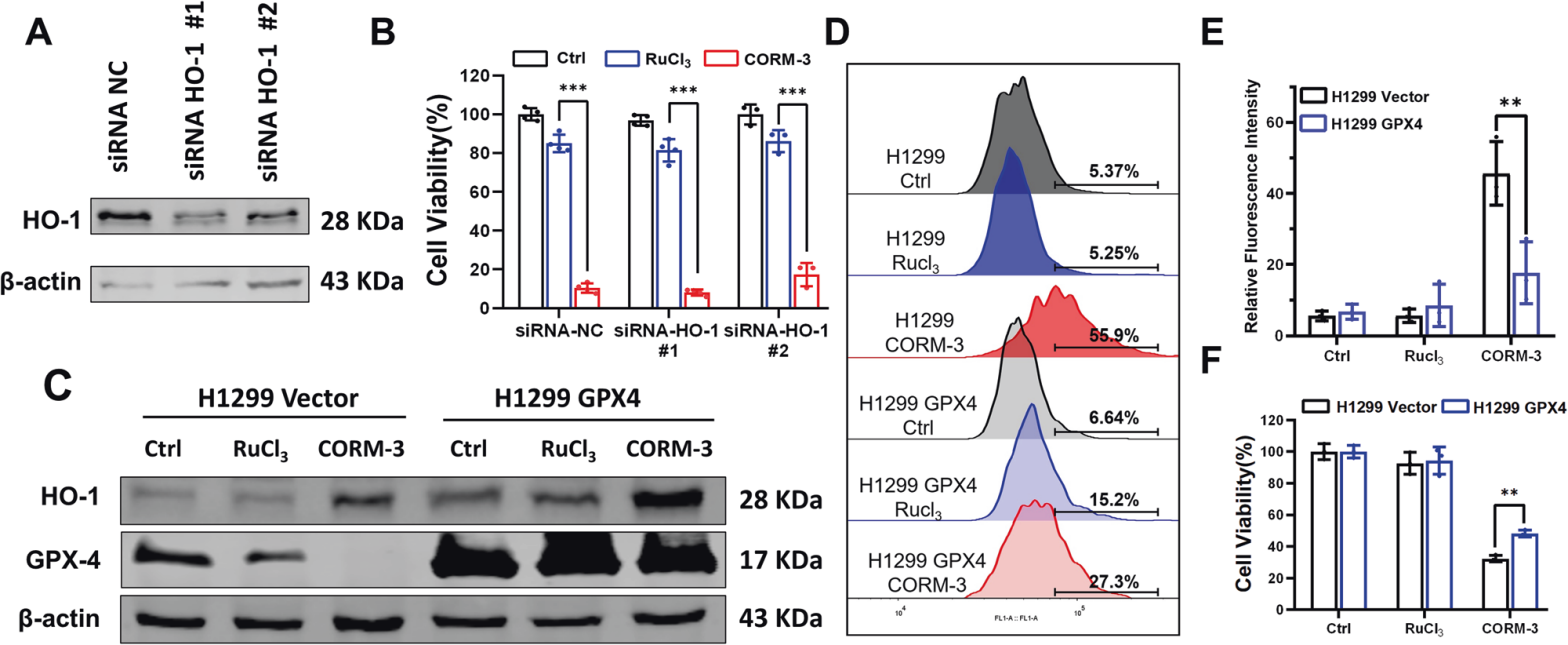
CO induces ferroptosis through GPX4 downregulation and ROS production. **A** Efficiency of knocking down HO-1 in H1299 cells detected with western blot. **B** Effect of HO-1 knockdown on the viability of H1299 cells treated with CORM-3. **C** Expressions of HO-1 and GPX4 in H1299/Vector and H1299/GPX4 cells at 72 h after CORM-3 treatment. **D**–**F** Levels of lipid peroxidation (**D**, **E**) and cell viability (**F**) in H1299-vector and H1299-GPX4 cells after CORM-3 treatment. *P < 0.05, **P < 0.01, ***P < 0.001.

Numerous evidences have indicated that upregulation of HO-1 and downregulation of GPX4 is involved in ferroptosis induction [21, 26]. To further confirm the critical role of HO-1 in CO-induced ferroptosis, HO-1 specific siRNA was employed to interfere with HO-1 expression in H1299 cells (Fig. 4A). Unfortunately, knockdown of HO-1 did not rescue the cell viability after CO treatment (Fig. 4B), suggesting that CO-induced ferroptosis was independent of HO-1. Meanwhile, GPX4-overexpressing H1299 cell line (H1299-GPX4) was constructed to investigate the role of GPX4 in CO-induced ferroptosis. Compared with the H1299-vector cells, both the H1299-GPX4 cells with or without CORM-3 treatment displayed enhanced expression of GPX4 (Fig. 4C), which indicated that the decrease of GPX4 induced by CO was rescued by overexpressing GPX4 (Fig. 4F). As expected, overexpression of GPX4 mitigated cell death and decreased the accumulation of lipid peroxidation after CORM-3 treatment (Fig. 4D, E), suggesting that GPX4 inhibition was a major contributor to CO-induced ferroptosis.

### CO down-regulates GPX4 expression *via* the activation of GSK3β

**Fig. 5:**
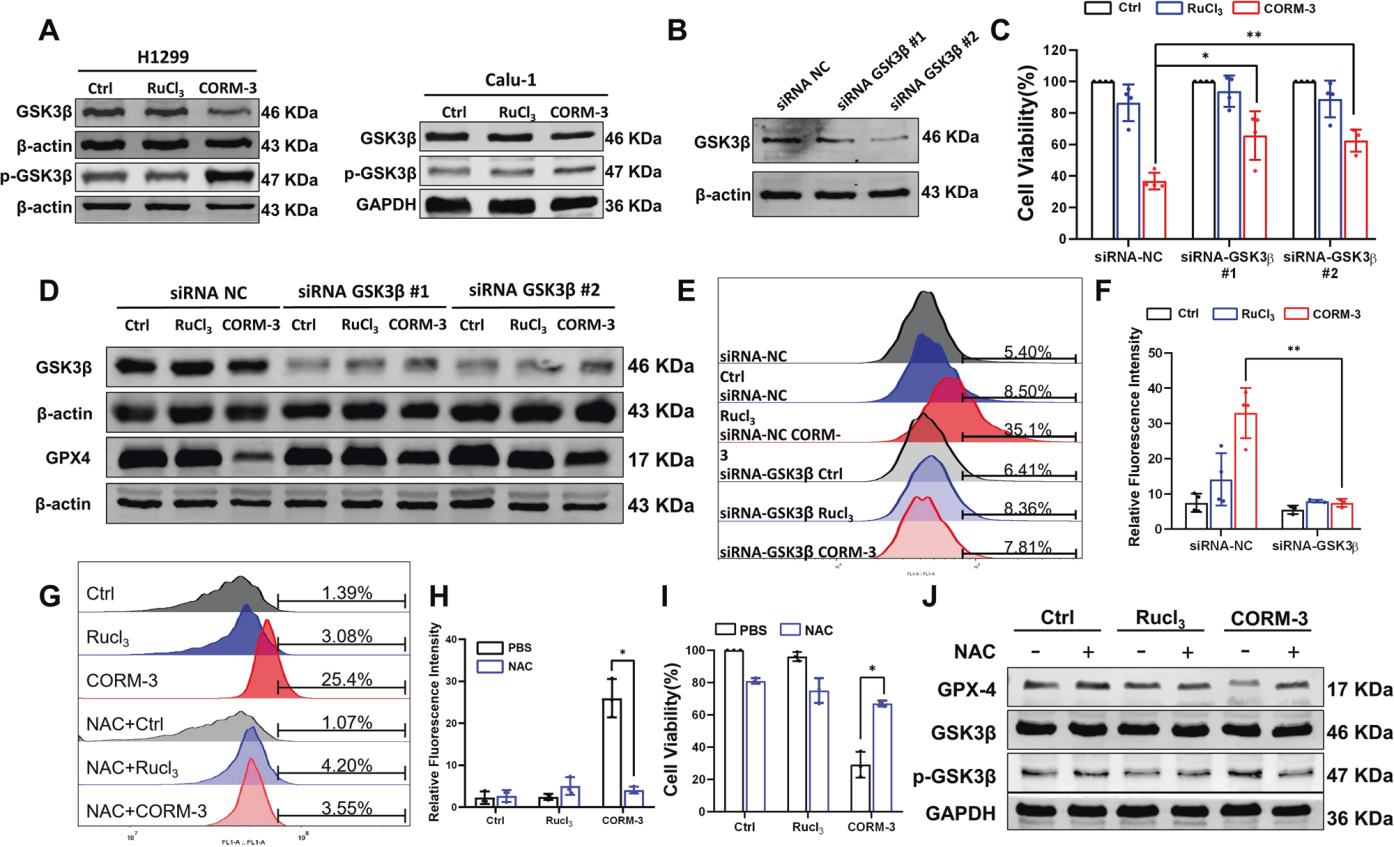
Activation of GSK3β suppressed the GPX4 expression after CORM-3 treatment. **A** Protein expressions of GSK3β and p-GSK3β in H1299 and Calu-1 cells at 72 h after CORM-3 treatment. **B** Efficiency of GSK3β knockdown detected with western blot. **C** Effect of GSK3β knockdown on the viability of H1299 cells treated with CORM-3. **D** Effect of GSK3β knockdown on the expression of GPX4 in H1299 cells after CORM-3 treatment. **E**, **F** Levels of lipid peroxidation determined by C11-BODIPY 581/591 (10 µM) in GSK3β-knockdown H1299 cells treated with CORM-3. **G**–**I** Effect of NAC (5 μM) on the levels of lipid peroxidation (**G**, **H**) and cell viability (**I**) in H1299 cells treated with CORM-3. **J** Protein expression of GPX4 and p-GSK3β in H1299 cells treated with CORM-3 in the presence of 5 μM NAC. *P < 0.05, **P < 0.01.

Interestingly, we found that activation of GSK3β, one critical regulator of GPX4 expression, was enhanced after treatment with CO in H1299 and Calu-1 cells (Fig. 5A). To further determine the possible involvement of GSK3β in CO-induced ferroptosis, GSK3β specific siRNA was employed to interfere with the expression of GSK3β in H1299 cells (Fig. 5B). As shown in Fig. 5C, GSK3β knockdown significantly increased the viability of H1299 cells treated with CORM-3, indicating the involvement of GSK3β in CO-induced ferroptosis. Furthermore, GSK3β knockdown dramatically alleviated the decrease of GPX4 protein expression after CORM-3 treatment (Fig. 5D), suggesting regulation of GPX4 by GSK3β. Similarly, accumulation of lipid peroxidation in response to CORM-3 treatment was also attenuated by knocking down GSK3β (Fig. 5E, F). The above results indicate that activation of GSK3β suppresses the expression of GPX4 and thus triggers ferroptosis. Moreover, NAC treatment nearly completely abrogated CO-induced cell death and accumulation of lipid peroxidation (Fig. 5G–I). The activation of GSK3β and decrease of GPX4 expression induced by CO were also not observed after NAC treatment (Fig. 5J), indicating that ROS might be the primary cause of tumor-cell ferroptosis in response to CO treatment.

### CO inhibits the growth of human lung cancer xenografts

**Fig. 6:**
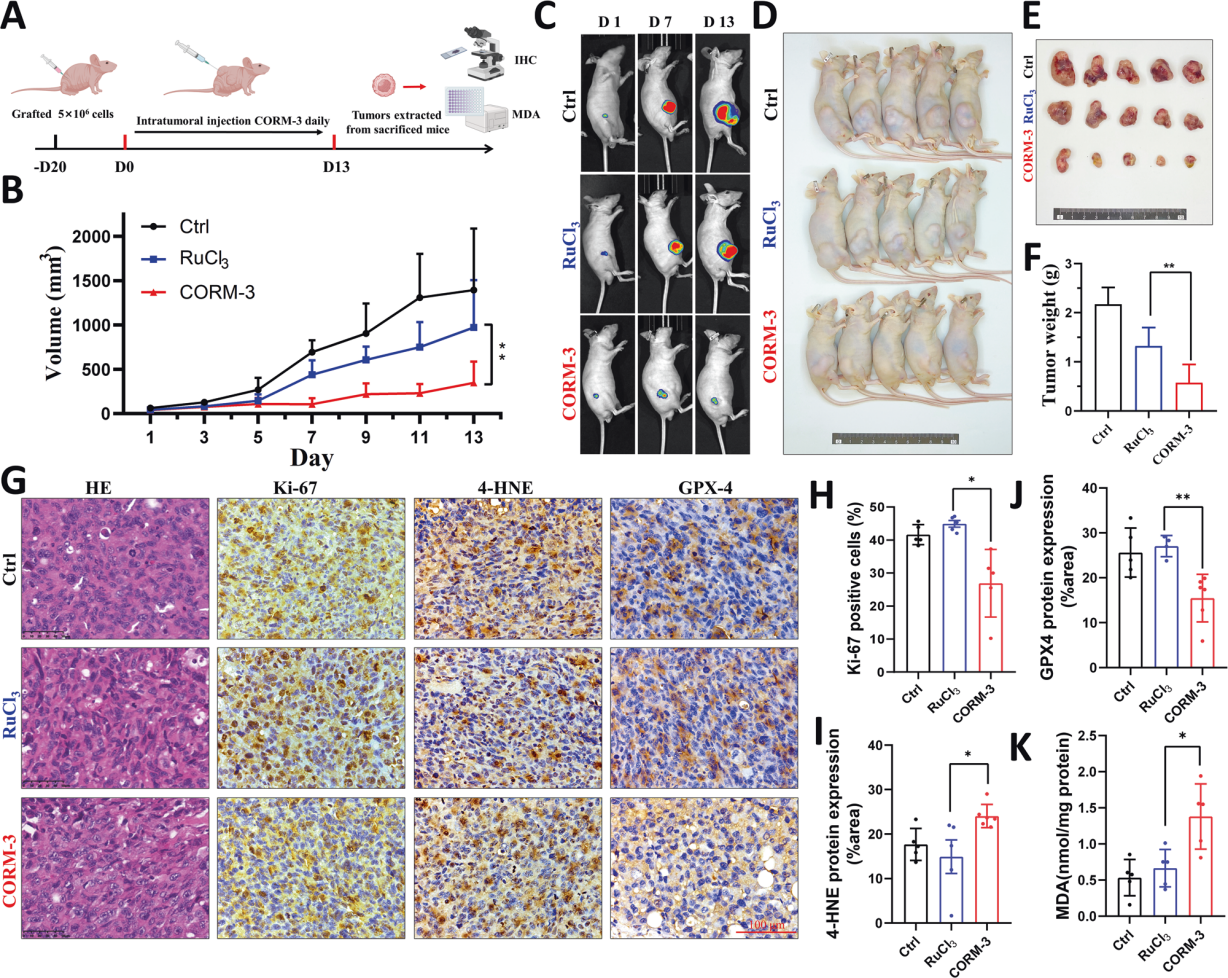
CO inhibits the growth of H1299-Luc tumor xenografts. **A** Schematic for experimental design. **B** Growth curves for H1299-Luc tumors in mice treated with CORM-3 or RuCl_3_. **C** Representative bioluminescence images of H1299-Luc tumors captured on days 1, 7 and 13 after CORM-3 or RuCl_3_ injections. **D** Macroscopic appearance of H1299-Luc tumors-bearing mice 13 days after CORM-3 or RuCl_3_ treatment. **E**, **F** Gross appearances (**E**) and tumor weights (**F**) of excised tumors from mice sacrificed at 13 days after intratumoral injection with CORM-3 or RuCl_3_. **G** Representative IHC images of Ki-67, 4-HNE and GPX4 in tumors cut from mice. Scale bar: 100 μm. **H**–**J** Quantification of Ki-67 (**H**), 4-HNE (**I**) and GPX4 (**J**) protein expression. **K** The level of MDA in H1299-Luc tumors with CORM-3 or RuCl_3_ treatment. *P < 0.05, **P < 0.01.

We further investigated the anti-tumor effect of CO in nude mice bearing H1299-Luc xenografts. Tumor-bearing mice were injected with either RuCl_3_ or CORM-3 daily for 13 days as depicted in Fig. 6A. Compared with the results of the RuCl_3_ treatment, significant suppression of tumor growth became apparent about 7 days after CORM-3 treatment, and the inhibition became more distinctly with the time (Fig. 6B, C). At the end of experiment, a significant reduction of tumor volume and weight in CORM-3 treatment group was observed compared with the RuCl_3_ treatment group (Fig. 6D–F), indicating that CO exerts anti-tumor activity in vivo, which was also supported by the decrease of Ki-67 proliferative index after CORM-3 treatment (Fig. 6G, H). Furthermore, we found CORM-3 treatment markedly elevated the levels of intratumoral MDA and 4-hydroxynonenal (4-HNE) (Fig. 6I, K), the secondary products of lipid peroxidation and the markers of ferroptosis. Consistent with our results in vitro, the expression of GPX4 protein in tumor was also down-regulated after treatment with CORM-3 but not RuCl_3_ (Fig. 6G, J). Taken together, these results indicate that CO treatment effectively induce ferroptosis and suppress tumor growth in vivo *via* decreasing GPX4 expression.

## Discussion

Recently, CO was found to have potential antitumor effects [5]. Accumulating evidences indicate that ROS and mitochondria have been implicated as the cellular targets for CO action [27–29]. Wegiel et al. showed that CO inhibited tumor growth by targeting mitochondria and promoting ROS generation [30]. This agrees with our results that CO treatment made mitochondrial morphology changes (shrunken mitochondria with disrupted cristae) and increased ROS production. It is noteworthy that CO also plays a negative role in ROS generation in previous studies. In osteoarthritic synoviocytes, CORM-2 attenuates ROS generation and exerts an anti-inflammatory effect [31]. In human gastric cancer cells, CORM-2 also abrogates IL-1β-induced ROS production [32]. These suggest that CO functioning as a ROS inducer or ROS scavenger might be dependent on the cell types [3]. Accordingly, further investigations are required to identify the relationship between CO and ROS generation.

In our study, we found that CO treatment induced increased HO-1 and decreased GPX4 expression, which have been previously reported to trigger ferroptosis. In fact, HO-1 has dual roles in ferroptosis induction. As a negative regulator of ferroptosis induction, enhanced HO-1 expression prevents ferroptosis induced by glutamate in HT-22 cells [33]. Conversely, HO-1 enhances the ferroptosis induced by Bay117085 *via* promoting iron accumulation and ROS generation [34]. However, our results showed that knocking down HO-1 had no effects on CO-induced cell death, suggesting neither a pro-death nor pro-survival role of HO-1 in CO-induced ferroptosis. We speculate that there should be other factor(s) involved in iron metabolism to induce ferroptosis by developing iron overload. As a hydroperoxide scavenger which converts lipid hydroperoxides to non-toxic lipid alcohols, GPX4 prevents ferroptosis by suppressing lipid hydroperoxides. Some chemotherapeutic drugs including apatinib [35], capsaicin [36], paclitaxel [37], etc., trigger ferroptosis *via* inhibiting GPX4 expression in several cancer cell types. Similar to previous studies, we also confirmed that downregulation of GPX4 contributed to CO-induced ferroptosis. However, overexpressing GPX4 only partially rescued the CO-induced ferroptosis, indicating the involvement of other signaling pathways in CO-induced ferroptosis which would require more in-depth studies.

Although it is known that down-regulation of GPX4 promotes induction of ferroptosis, the upstream signal pathways modulating GPX4 expression are not yet fully understood. Several factors, such as ZEB1, Nrf2, ATF-4 and microRNA-15a, etc. [38–40], have been reported to regulate GPX4 expression. A previous work indicates that that CBS is responsible for the downregulation of GPX4 induced by CO in MCF-7 cells [41]. In our study, inhibition of CBS was not observed after CO treatment in H1299 and Calu-1 cells, suggesting the independence of CBS activity in ferroptosis induction (Supplementary Fig. S1). Our findings further confirmed that activation of GSK3β after CO exposure induces ferroptosis *via* down-regulating GPX4 expression, and this is different from a previous result that activation of GSK3β promotes ferroptosis via dominating iron homeostasis in Hela cells [42]. These findings suggest that GSK3β might regulate different the signal pathways to induce ferroptosis in various cell lines.

**Fig. 7:**
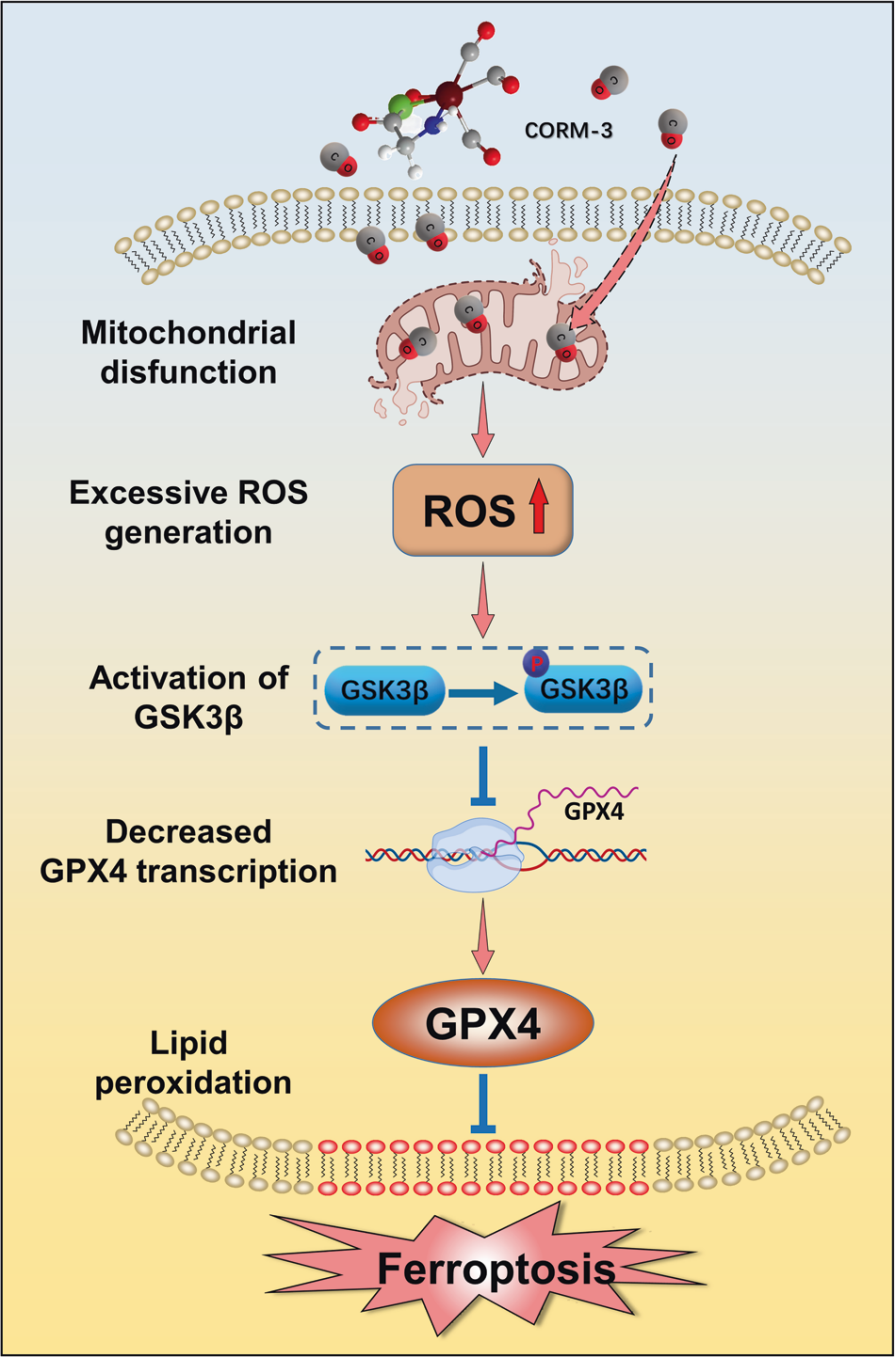
Schematic diagram of CO-induced ferroptosis. CO increased the mitochondrial ROS production; the increased level of intracellular ROS promoted activation of GSK3β; GSK3β activation inhibited the transcription of GPX4, and then enhanced the accumulation of lipid peroxidation, resulting in ferroptosis.

In summary, low concentration of exogenous CO induced ferroptosis in a time- and dose-dependent manner in NSCLC cells rather than normal lung cells. Mechanistically, we found that CO treatment activated the ROS/GSK3β/GPX4 signaling pathway, then led to a dramatic decrease in GPX4 expression and the accumulation of lipid peroxides and induced ferroptosis finally (Fig. 7). We hope CO, identified as a novel ferroptosis inducer, may be applied as a potential chemosensitizer to overcome the resistance caused by apoptosis-inducing chemotherapy drugs in the future.

## Materials and methods

### Cell culture

H1299, Calu-1 and HEK-293T cells were obtained from the Cell Bank of Type Culture Collection of the Chinese Academy of Sciences (Shanghai, China). The H1975 cell line was purchased from the American Type Culture Collection (Manassas, USA). Calu-1, H1299 and H1975 cells were cultured with RPMI-1640 medium (Gibco, Carlsbad, USA). HEK-293T cells were cultured with DMEM medium (Gibco, CA, USA). All the culture media were supplemented with 10% FBS (Lonsera, Montevideo, Uruguay), 100 µg/mL streptomycin and 100 U/mL penicillin. All cells were authenticated by STR profiling recently and verified to be free of mycoplasma and cultured at 37 °C in 5% CO_2_.

### Reagents and antibodies

CORM-3 (S7448), one kind of water soluble CO-releasing molecules, was obtained from Selleck (Selleck Chemicals, Shanghai, China). RuCl_3_, the negative control for CORM-3, was produced by completely releasing CO from CORM-3. Ferrostatin-1 (HY-100579), Liproxstatin-1 (HY-12726), Necrostatin-1 (HY-15760), Z-VAD-fmk (HY-16658B) and were purchased from MCE (MedChemExpress, New Jersey, USA).

The primary antibodies used in the present study including anti-GPX4 (52455), anti-HO-1 (43966S), and anti-p-GSK3β (9322 S) were purchased from CST (Cell Signaling Technology, Beverly, USA). Anti-GAPDH (60004-1-Ig), Anti-Ki-67 (27309-1-AP) and anti-β-actin (66009-1-Ig) antibodies were obtained from Proteintech (Wuhan, China). The antibody of anti-rabbit-GSK3β (WL01456) and anti-mouse-GSK3β (221162) antibodies were obtained from Wanleibio (Shenyang, China). The antibody of anti-4-HNE (mab3249) was obtained from Bio-Techne (Minneapolis, USA). The secondary antibodies, including anti-rabbit IRDye800® conjugated antibody (926-32211) and anti-mouse IRDye®680DX conjugated antibody (926-68070), were purchased from Li-COR (LI-COR Biosciences, Lincoln, USA).

### Cell viability assay

Cell viability was measured with Cell Counting Kit-8 (CCK-8) assay (K1018, ApexBio, Houston, USA) according to the manufacturer’s recommendation. Cell viability (%) = [(Absorbance of tested compound minus Absorbance of blank)/(Absorbance of control minus Absorbance of blank)] × 100%.

### Colony formation assay

The cells were plated into a 35 mm cell culture dish at a density of 300 cells/dish followed by CORM-3 treatment. After 10 days incubation, the cells were fixed with 4% paraformaldehyde and stained with 1% crystal violet. The colonies containing ≥50 cells were counted and the survival fraction was calculated for each group.

### Soft agar colony formation assay

The prepared 0.6% agar in complete growth medium was plated in six-well plates as the base agar layer. After 30 min, re-suspended cells (1000 cells/mL) in the growth medium containing 0.36% agar were seeded on the top of the solidified base layer, and then incubated at 37 °C and 5% CO_2_. After 2 weeks incubation, the number of colonies was counted with microscopy.

### Western blotting

The cells were lysed in RIPA buffer (P0013B, Beyotime Biotechnology, Shanghai, China) with PMSF (1 mM, ST506, Beyotime Biotechnology, Shanghai, China). The protein extracts were subjected to SDS-PAGE and transferred onto polyvinyl difluoride (PVDF) membranes. Subsequently, the membranes were incubated with primary antibodies at 4 °C overnight after blocking with 5% nonfat dry milk for 1 h at room temperature. After washing three times with TBST (0.1% Tween-20 in Tris-HCl buffer), the membranes were incubated with IRDye-labelled secondary antibodies for 1 h at room temperature, and the infrared fluorescence signals were detected with an Odyssey® CLx Infrared Imaging System (9140-00, Li-COR Biosciences, Lincoln, USA).

### RNA extraction, mRNA sequencing (mRNA-seq) and quantitative real-time PCR

Total RNA was isolated with RNeasy Mini kit (74004, Qiagen, Hilden, Germany) and cDNAs were synthesized from 2 μg total RNA with NovoScript 1st Strand cDNA Synthesis kit (E047-01B, Novoprotein, Shanghai, China).

Library preparation and data analysis of high throughput sequencing were conducted by Kangce Technology Co., Ltd (Wuhan, China). RNA-seq library was constructed by Illumina TruSeq RNA Sample Prep Kit version 2 (Illumina, San Diego, CA) and RNA was sequenced by Illumina HiSEquation 2000 platform (Illumina). A gene is considered differentially expressed (DEG) if it has an FDR step-up *p* ≤ 0.05 and a fold-change ≥ ±2. KEGG pathway analyses (https://www.kegg.jp/entry/map04216) were performed by using the DAVID website to study the function of DEGs. Values with *p* < 0.05 were considered statistically significant.

Real-time PCR (qPCR) was performed with Hieff qPCR SYBR Green Master Mix Kits (11203ES03, Yeasen, Shanghai, China) on a Roche 480 Light Cycler (05015278001, Roche, Basel, Switzerland). The primers for PCR amplification were shown as follows: 5’-GAGGC AAGACCGAAGTAAACTAC-3’, 5’-CCGAACTGGTTACACGGGAA-3’ (GPX4); 5’-AAGACTGCGTTCCTGCTCAAC’, 5’-AAAGCCCTACAGCAACTGTCG-3’ (HO-1); and 5’-GGAGCGAGATCCCTCCAAAAT-3’, 5’-GGCTGTTGTCATACTTCTCATGG-3’ (GAPDH). The GAPDH gene was used as a control, and the data were presented as fold changes of gene expression in the tested samples compared to the control with the 2^-ΔΔCt^ method.

### Gene set enrichment analysis (GSEA)

Gene set enrichment analysis (GSEA) was performed to clarify the enriched KEGG pathways by using the gene set “c2. cp.kegg.v7.4. symbols.gmt” as a reference. Normalized enrichment score (NES) absolute value greater than 1, false discovery rate (FDR) *q* < 0.25 and *P* < 0.05 were used as the criteria in defining the significant difference.

### Construction of protein-protein interaction (PPI) network

STRING database (http://string-db.org/) and Cytoscape software were used in the process of drawing the PPI networks of ferroptosis-related genes. Gene sets obtained by Venn analysis were inserted into STRING to generate PPI diagram, and the degree plugin was applied to screen pivotal genes.

### Malondialdehyde (MDA) detection

The MDA level was assessed with a lipid peroxidation MDA Assay Kit (S0131S, Beyotime Biotechnology, Shanghai, China) following manufacturer’s instructions. The cells were lysed with the lysis buffer and the isolated tumors were ground and lysed to obtain the tumor homogenate. After centrifugation, the supernatants were mixed with thiobarbituric acid (TBA) and incubated at 95 °C for 1 h. The absorbance was measured with a microplate reader at 532 nm to evaluate the MDA concentration.

### Flow cytometry for lipid peroxidation detection

The cells were collected and washed with PBS at 72 h after treatment. Subsequently, the cells were re-suspended in medium without FBS and stained with C11 BODIPY 581/591 (D3861, Invitrogen, Carlsbad, USA) for 30 min, followed by flow cytometry analysis. Raw data were then processed with the FlowJo software.

### Transmission electron microscopy

The cells were fixed with 2.5% glutaraldehyde in phosphoric acid buffer for 2 h. After dehydration through an ethanol series, cells were embedded and then sectioned followed by staining with 3% uranium acetate and lead citrate. The images were then captured with a transmission electron microscope (JEM-1011, JEOL Ltd, Tokyo, Japan).

### Establishing stable GPX4-overexpressing cell

Human GPX4 plasmid was constructed by inserting GPX4 cDNAs (NM_001039847.3) into a pCDH lentiviral vector (CD810A-1, Miaolingbio, Wuhan, China). To generate lentiviruses, the constructed plasmid was transfected into HEK-293T cells with pSPAX2 and pMD2G at a ratio of 5:3:2. Lentivirus-containing supernatant collected at 48 h after transfection was used to infect H1299 cells. At 48 h after infection, the cells were selected with 2 μg/mL puromycin for 14 days to obtain the GPX4-overexpressing H1299 cells (H1299-GPX4). To detect the tumor volumes in vivo, H1299 cells were stably expressed firefly luciferase by transfecting with pLentipuro3-TO-V5-GW-EGFP-Firefly Luciferase plasmid (P21289, Miaolingbio, Wuhan, China).

### Small interfering RNA transfection

The siRNAs for HO-1 and the negative control siRNA were synthesized by GenePharma (Shanghai, China). The siRNA sequences of HO-1 were: siRNA#1, CCAGGCAGCTGATCATAGA; and siRNA#2, CGACCTGACTGCCAAGAAA. The siRNA sequences of GSK3β were: siRNA#1, CCACAAGAAGUCAGCUAUA; and siRNA#2, GGAU-CAGUUGGUAGAAAUA. The cells were transfected with double-stranded siRNAs by using the Lipofectamine®2000 transfection reagent (11668019, Thermo Fisher scientific, San Diego, USA) according to the manufacturer’s protocol.

### Tumor growth and treatments

Six-week-old male Balb/C-nude mice, obtained from GemPharmatech company (Nanjing, China), were grafted with 5 × 10^6^ cells (H1299-Luc) by subcutaneous injection into the right flank. When tumors reached a size of ~50 mm^3^, mice were randomized into three groups (*n* = 5) following random number table method and treated with PBS, RuCl_3_ (30 mg/kg) or CORM-3 (30 mg/kg) via intratumoral injection once daily. Tumor growth was monitored by measuring the volume of tumors every two days. No sample animals were excluded from this study. The study was performed in non-blinded modality.

The volume of a tumor was calculated as (L × W × H)/2, with the length L (the longest dimension), width W (the distance perpendicular to and in the same plane as the length), and height H (the distance between the exterior tumor edge and the mouse’s body) of each tumor was measured with vernier calipers. The mice were sacrificed at 13 days after CORM-3 treatment. Tumor xenografts were collected, photographed, and weighed and the ferroptosis was evaluated by immune-histochemistry (4-HNE) and MDA detection.

### HE staining and immune-histochemistry (IHC)

The tumor sections were deparaffined in xylene, rehydrated through gradient ethanol and then stained with H&E staining kit (Abcam, Cambridge, USA) as per manufacturers’ instructions. The expression levels of Ki-67, GPX4 and 4-HNE were detected with immunohistochemistry. The IHC results were scored as the percentage of positively stained cells by using Image *J* (IJ 1.46r).

### Statistical analysis

All experiments were performed at least three times. All analyses were performed with the GraphPad Prism 9 statistical software (GraphPad Software Inc., San Diego, USA). For all experiments, comparisons between two groups were based on *t* test, and one-way analysis of variance (ANOVA) was used to assess the difference between more than two groups. Data are shown as means ± SD. *P < 0.05, **P < 0.01 and ***P < 0.001; ns no significance.

### Supplementary information

Original Data File

Extended Data 1

## Data Availability

The data that support the findings of this study are available on request from the corresponding authors upon reasonable request.
