# Correction: Exogenous carbon monoxide promotes GPX4-dependent ferroptosis through ROS/GSK3β axis in non-small cell lung cancer

**DOI:** 10.1038/s41420-024-02086-0

**Published:** 2024-07-17

**Authors:** Wei Cao, Mingyu Sun, K. N. Yu, Lele Zhao, Yue Feng, Chunhua Tan, Miaomiao Yang, Ying Wang, Fengqin Zhu, Lianjun Chen, Lili Nie, Ye Zhao, Guodong Chen, Wei Han

**Affiliations:** 1 Anhui Province Key Laboratory of Medical Physics and Technology, Institute of Health and Medical Technology, Hefei Institutes of Physical Science, Chinese Academy of Sciences, 230031 Hefei, PR China; 2 Teaching and Research Section of Nuclear Medicine, School of Basic Medical Sciences, Anhui Medical University, 230031 Hefei, PR China; 3 Hefei Cancer Hospital, Chinese Academy of Sciences, 230031 Hefei, PR China; 4 Department of Physics, City University of Hong Kong, 999077 Hong Kong, PR China; 5 State Key Laboratory in Marine Pollution, City University of Hong Kong, 999077 Hong Kong, PR China; 6 School of Biology, Food and Environment, Hefei University, 230031 Hefei, PR China; 7 Collaborative Innovation Center of Radiation Medicine of Jiangsu Higher Education Institutions and School for Radiological and Interdisciplinary Sciences (RAD-X), Soochow University, 215006 Suzhou, PR China

**Keywords:** Non-small-cell lung cancer, Cell death

Correction to: *Cell Death Discovery* 10.1038/s41420-023-01743-0, published online 23 January 2024

In the published article, the affiliation order of Wei Han, the main corresponding author of the article, in the author list is: 1, 2, 3, 7.

It should read: “2, 3, 7”

The original article has been corrected.

