# Supplementary material for: Exogenous carbon monoxide promotes GPX4-dependent ferroptosis through ROS/GSK3β axis in non-small cell lung cancer
Source: Cell Death Discov. 2024 Jan 23;10:42. doi: 10.1038/s41420-023-01743-0 (PMC10805785; doi:10.1038/s41420-023-01743-0)

fig 3 G (H1299)

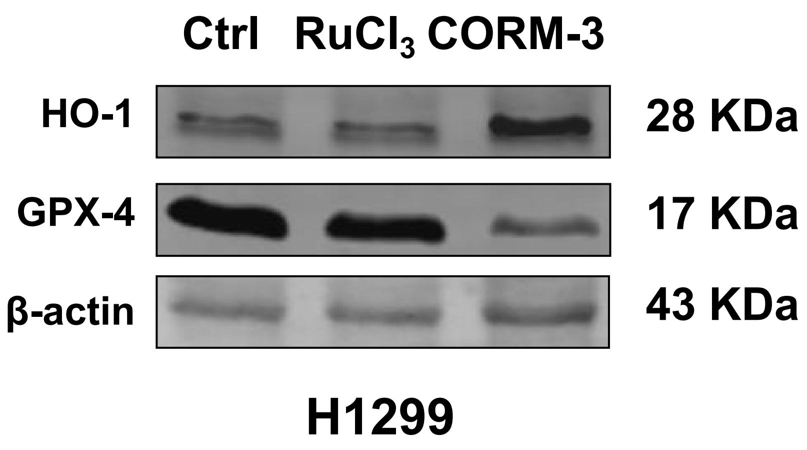
Ctrl
RuCl3 CORM-3
HO-1
GPX-4
β-actin
28 KDa
17 KDa
43 KDa
H1299

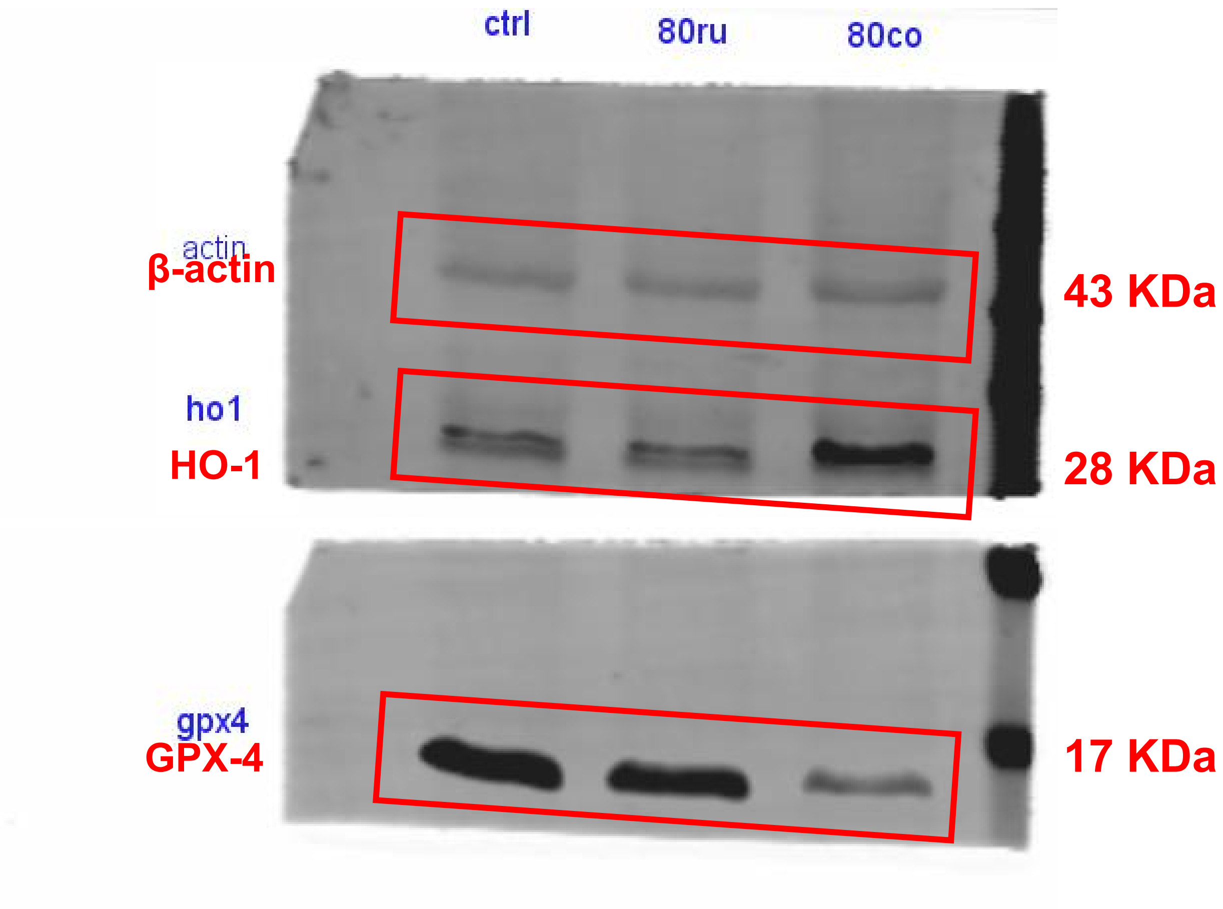
ctrl
80ru
80co
actin
β-actin
ho1
HO-1
gpx4
GPX-4
43 KDa
28 KDa
17 KDa

fig 3 G (Calu-1)

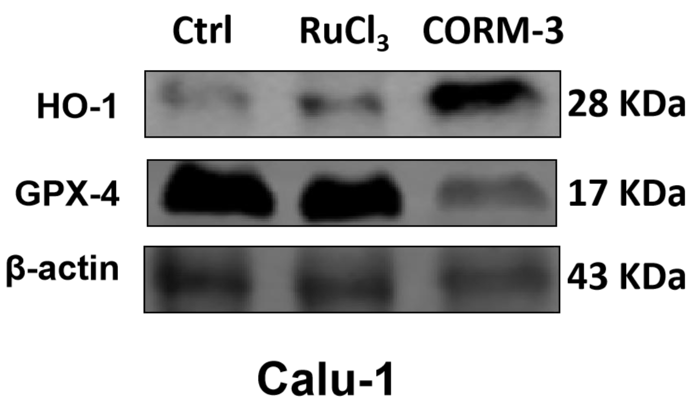
Ctrl
RuCl3
CORM-3
HO-1
GPX-4
β-actin
28 KDa
17 KDa
43 KDa
Calu-1

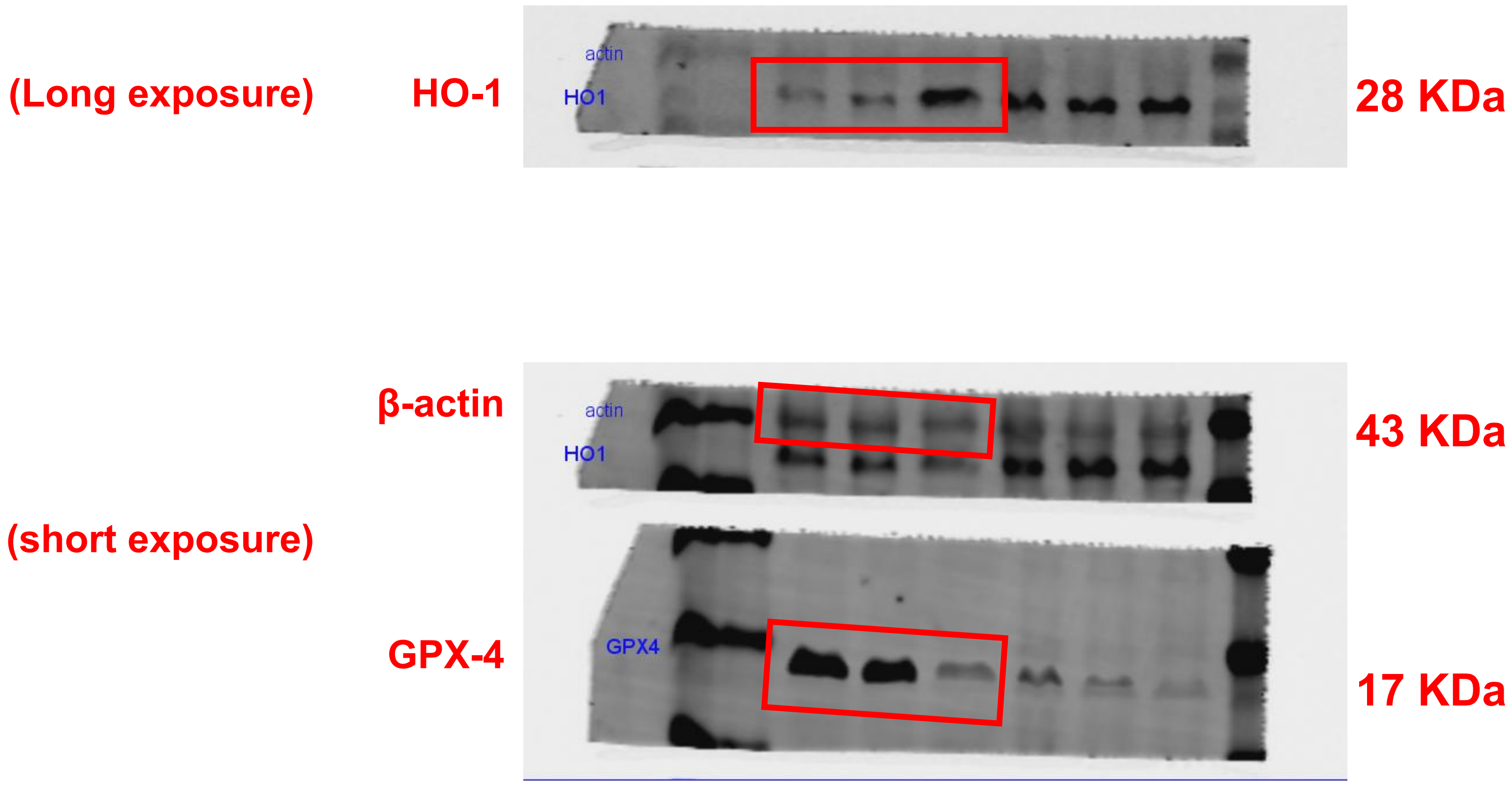
(Long exposure)
HO-1
actin
HO1
28 KDa
β-actin
(short exposure)
GPX-4
actin
HO1
GPX4
43 KDa
17 KDa

fig 4 A

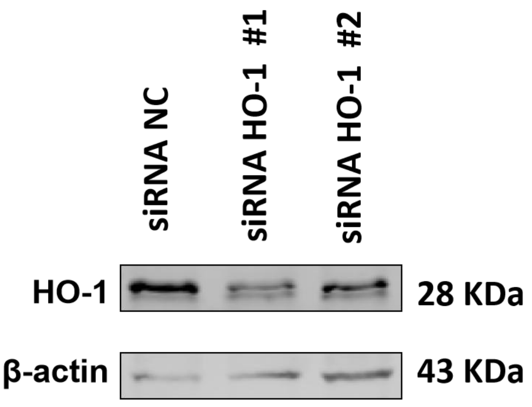

siRNA NC
siRNA HO-1 #1
siRNA HO-1 #2
HO-1
28 KDa
β-actin
43 KDa

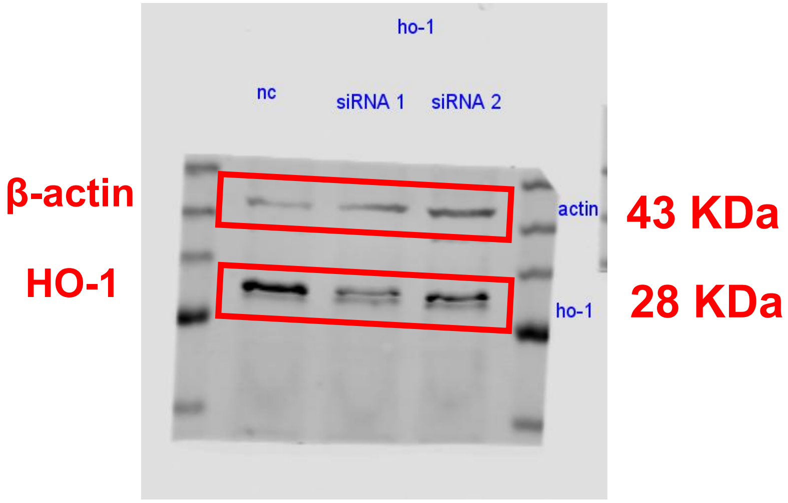

ho-1
nc
siRNA 1
siRNA 2
β-actin
actin
43 KDa
HO-1
ho-1
28 KDa

fig 4 C

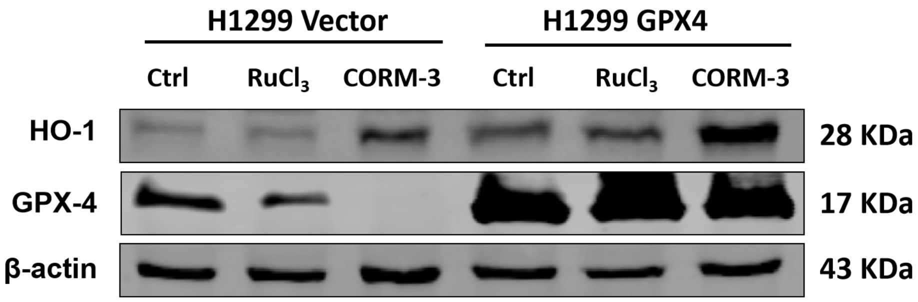

H1299 Vector
H1299 GPX4
Ctrl
RuCl3
CORM-3
Ctrl
RuCl3
CORM-3
HO-1
GPX-4
β-actin
28 KDa
17 KDa
43 KDa

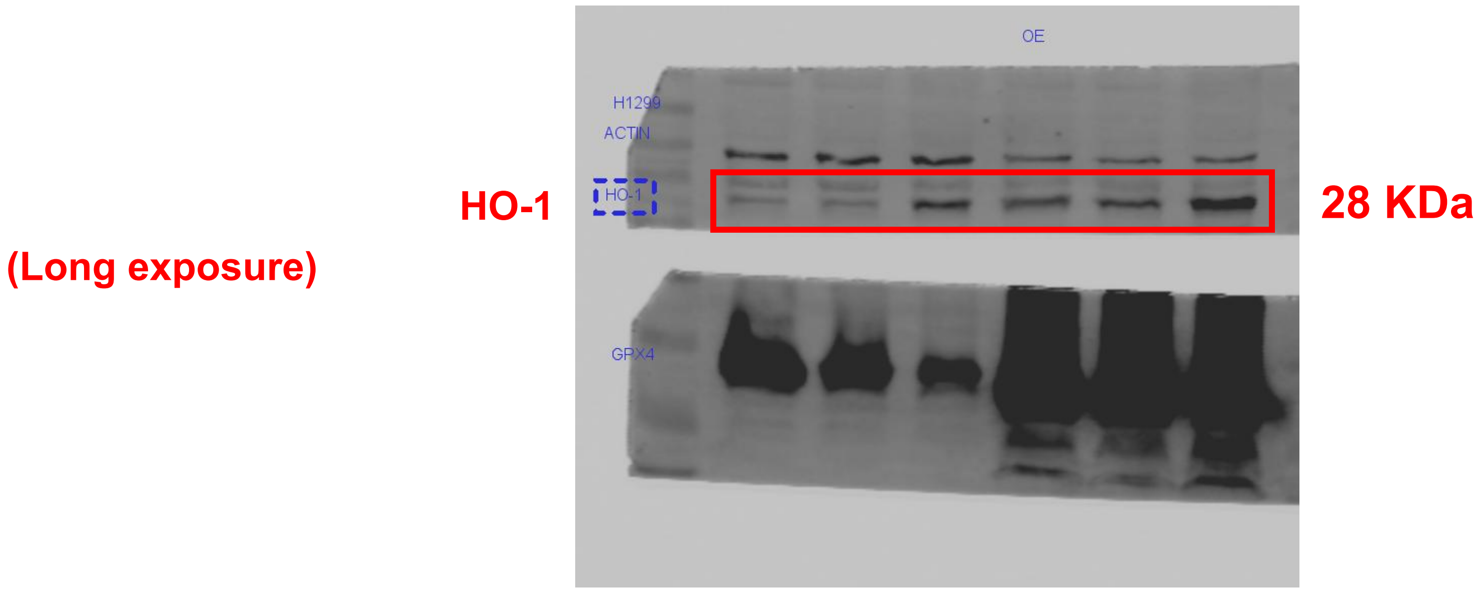

(Long exposure)
HO-1
28 KDa
OE
H1299
ACTIN
HO-1
GPX4

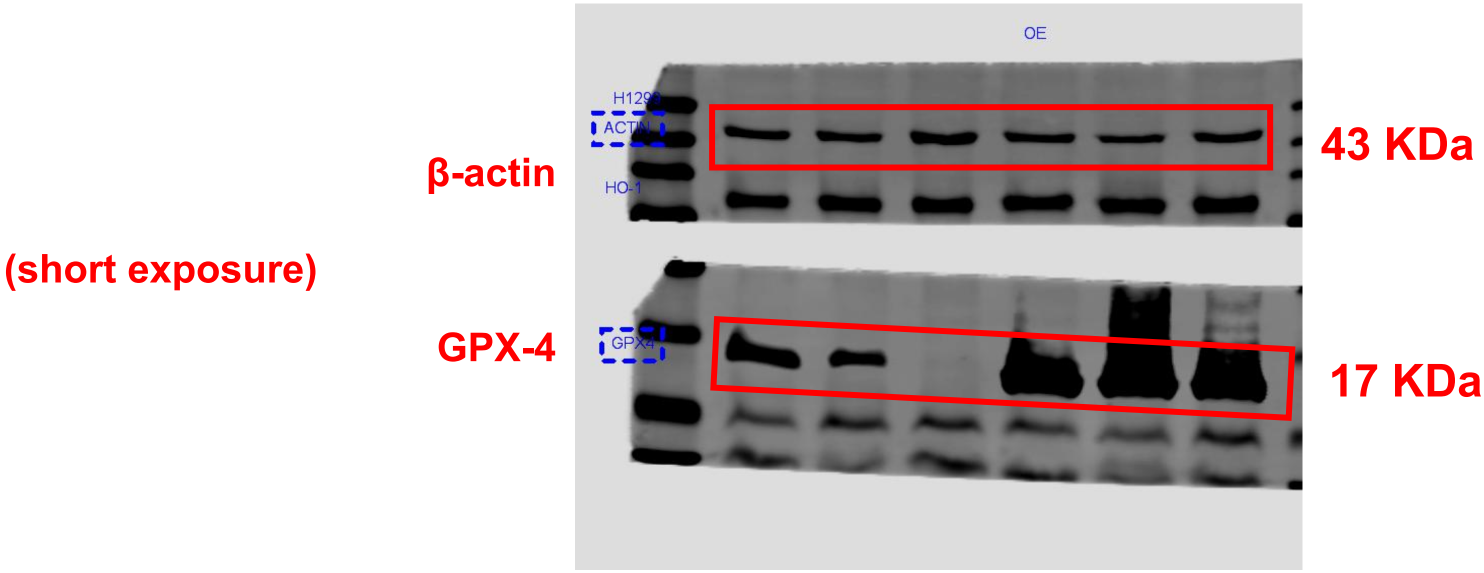

(short exposure)
β-actin
GPX-4
43 KDa
17 KDa
OE
H1299
ACTIN
HO-1
GPX4

fig 5 A (H1299)

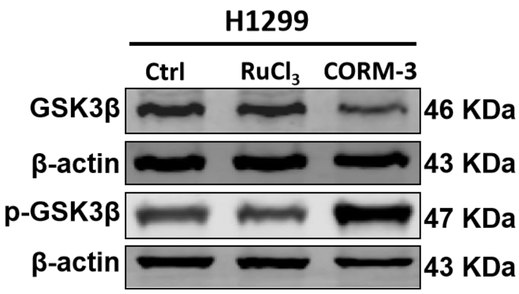

H1299
Ctrl
$RuCl_3$
CORM-3
GSK3β
β-actin
p-GSK3β
β-actin
46 KDa
43 KDa
47 KDa
43 KDa

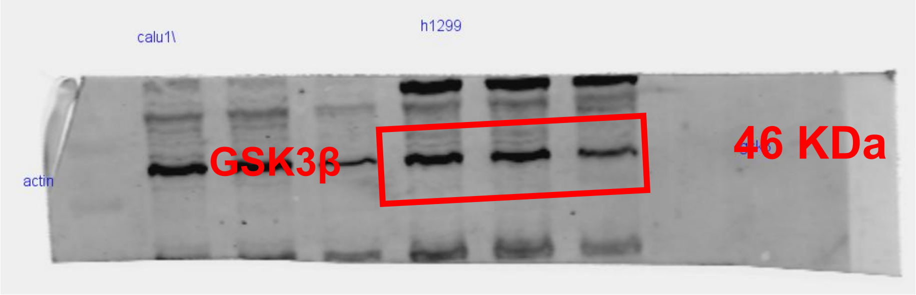

calu1\
h1299
actin
GSK3β
46 KDa

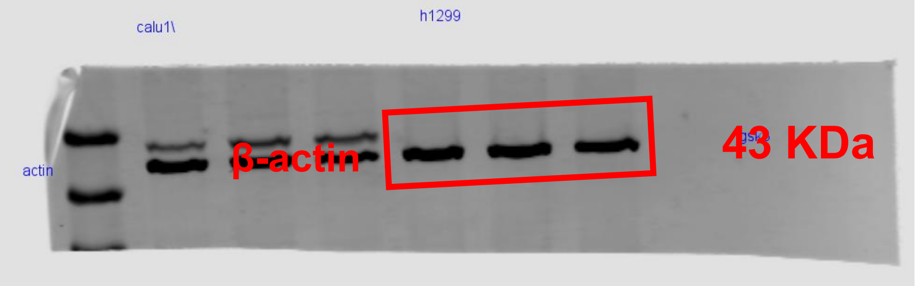

calu1\
h1299
actin
β-actin
43 KDa

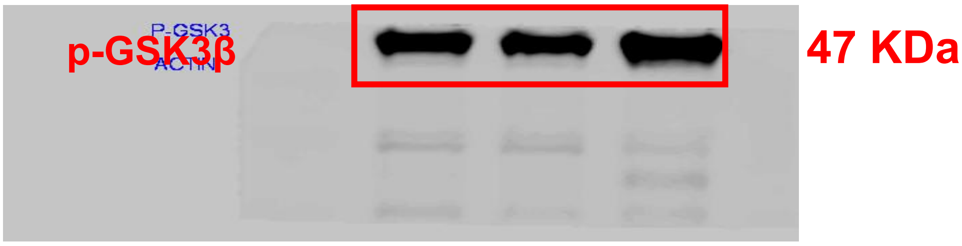

p-GSK3β
47 KDa

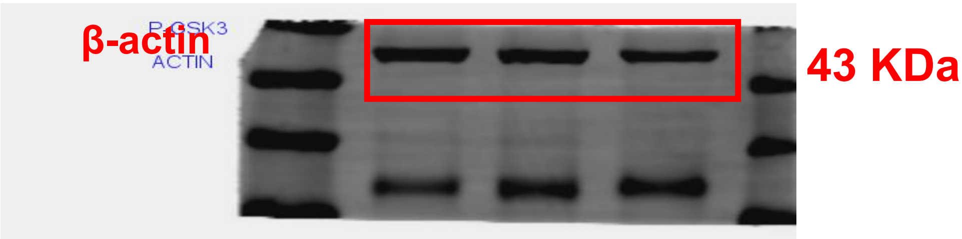

β-actin
ACTIN
43 KDa

fig 5 A (Calu-1)

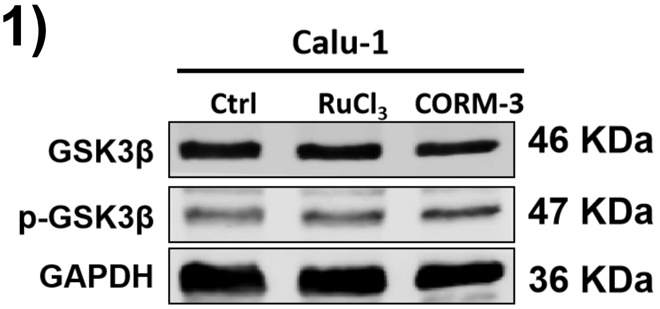
Calu-1
Ctrl
RuCl3
CORM-3
GSK3β
p-GSK3β
GAPDH
46 KDa
47 KDa
36 KDa

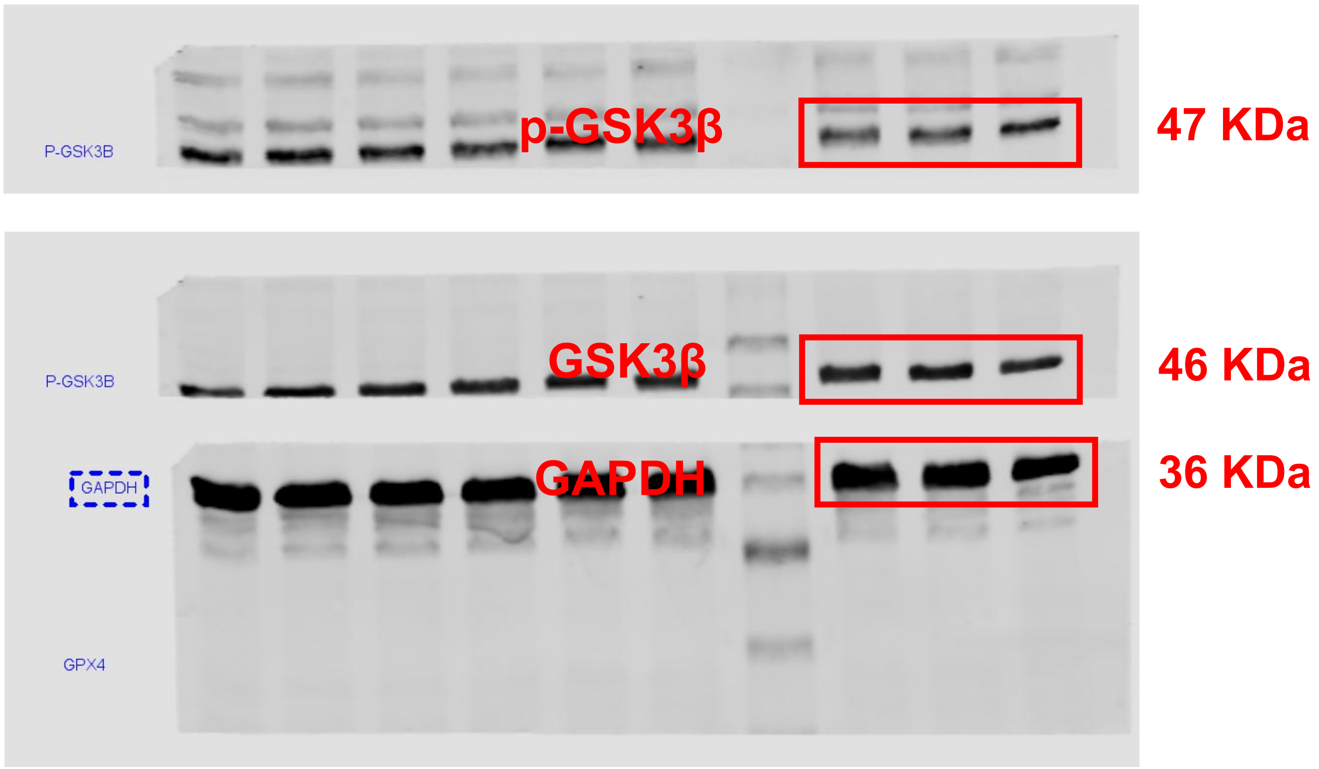
P-GSK3B
p-GSK3β
47 KDa
P-GSK3B
GSK3β
46 KDa
GAPDH
GAPDH
36 KDa
GPX4

fig 5 B

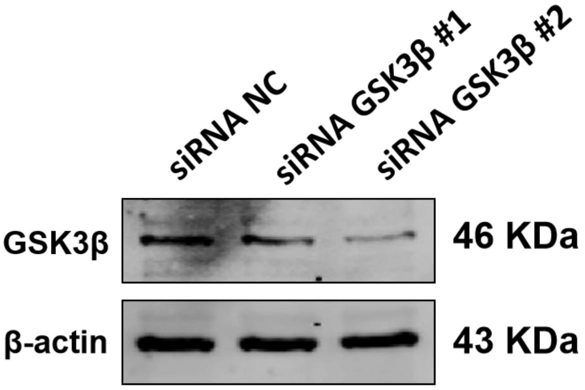

siRNA NC
siRNA GSK3β #1
siRNA GSK3β #2
GSK3β
β-actin
46 KDa
43 KDa

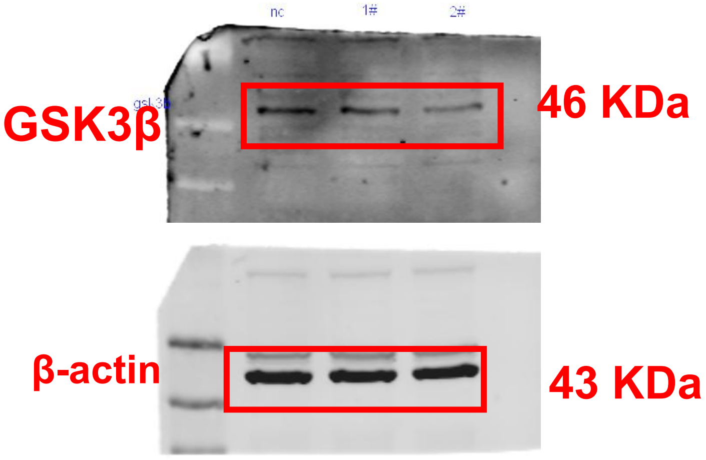

nc
1#
2#
GSK3β
46 KDa
β-actin
43 KDa

fig 5 D

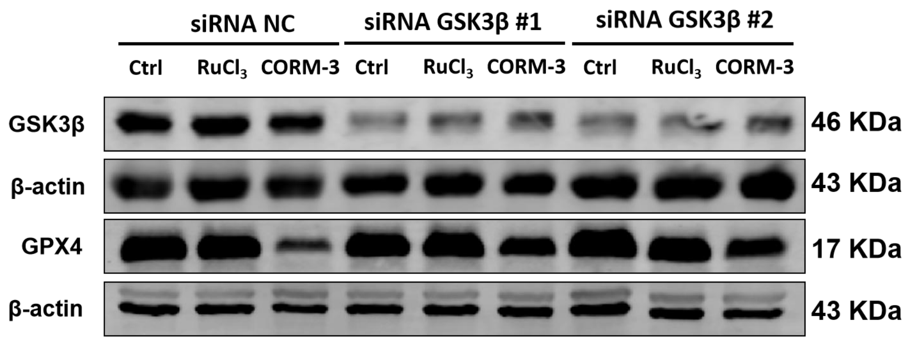
siRNA NC
siRNA GSK3β #1
siRNA GSK3β #2
Ctrl RuCl$_3$ CORM-3 Ctrl RuCl$_3$ CORM-3 Ctrl RuCl$_3$ CORM-3
GSK3β
β-actin
GPX4
β-actin
46 KDa
43 KDa
17 KDa
43 KDa

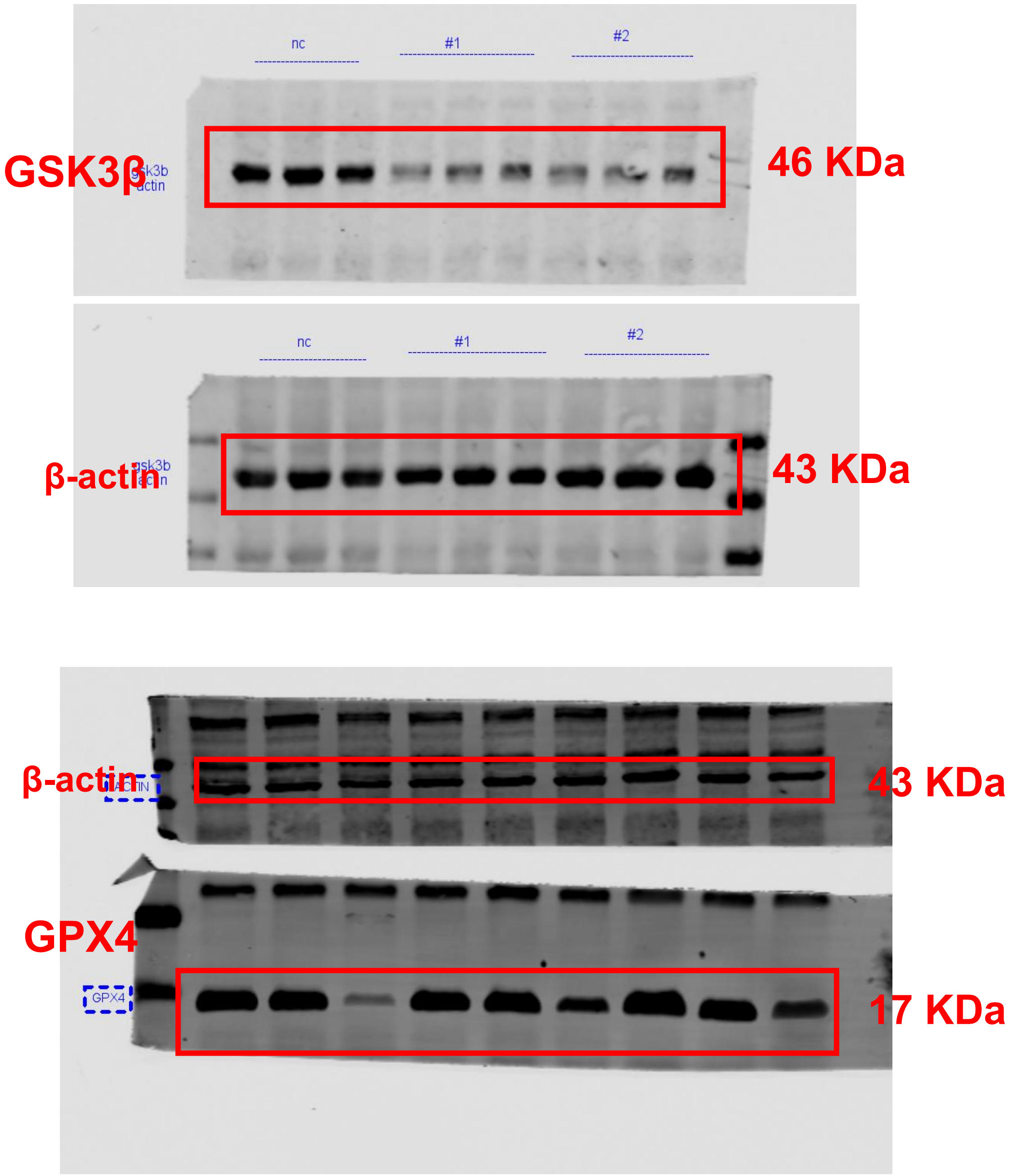
nc
#1
#2
GSK3β
46 KDa
β-actin
43 KDa
β-actin
43 KDa
GPX4
GPX4
17 KDa

fig 5 J

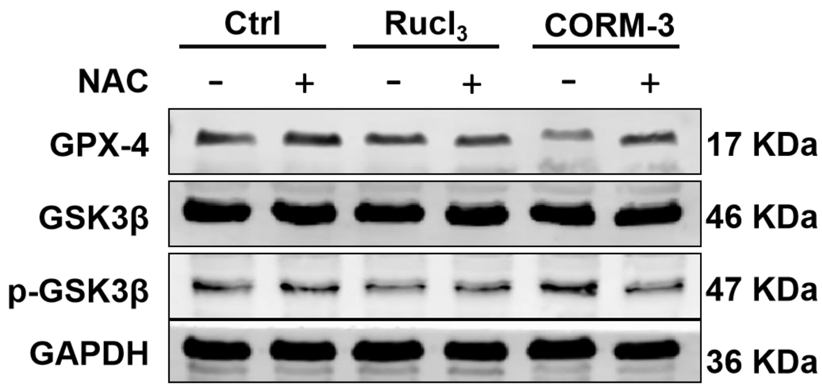

Ctrl
Rucl3
CORM-3
NAC
-
+
-
+
-
+
GPX-4
GSK3β
p-GSK3β
GAPDH
17 KDa
46 KDa
47 KDa
36 KDa

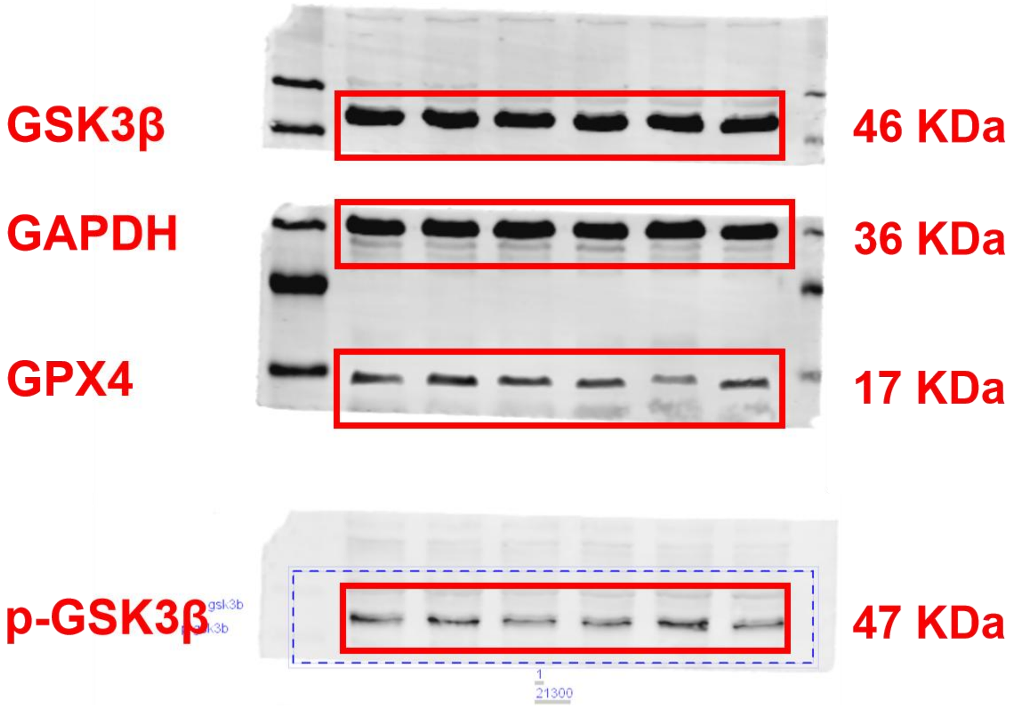

GSK3β
46 KDa
GAPDH
36 KDa
GPX4
17 KDa
p-GSK3β
47 KDa

Supplementary Fig. 1

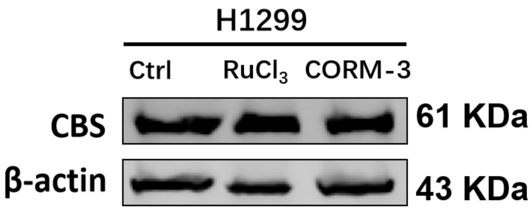

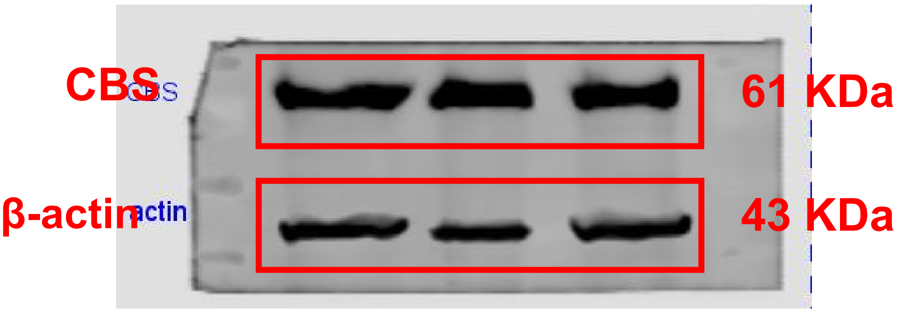

Supplementary Fig. 1

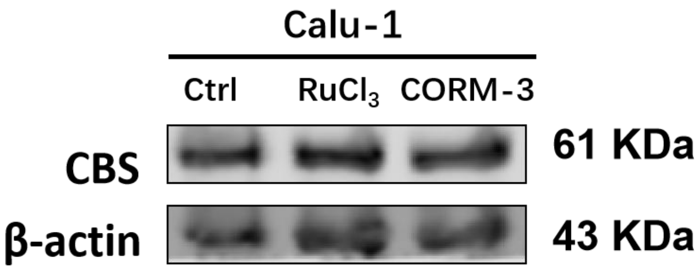

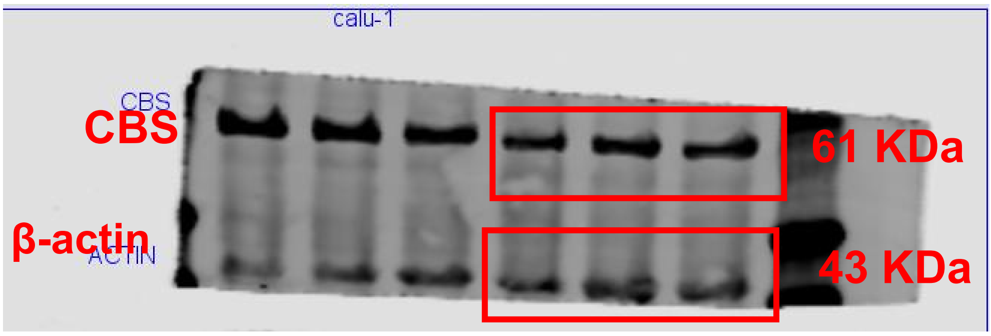

Supplement: Supplementary file 1 — Original Data File [file 41420_2023_1743_MOESM1_ESM.pdf]
