# Supplementary figures and images for: Exogenous carbon monoxide promotes GPX4-dependent ferroptosis through ROS/GSK3β axis in non-small cell lung cancer
Source: Cell Death Discov. 2024 Jan 23;10:42. doi: 10.1038/s41420-023-01743-0 (PMC10805785; doi:10.1038/s41420-023-01743-0)

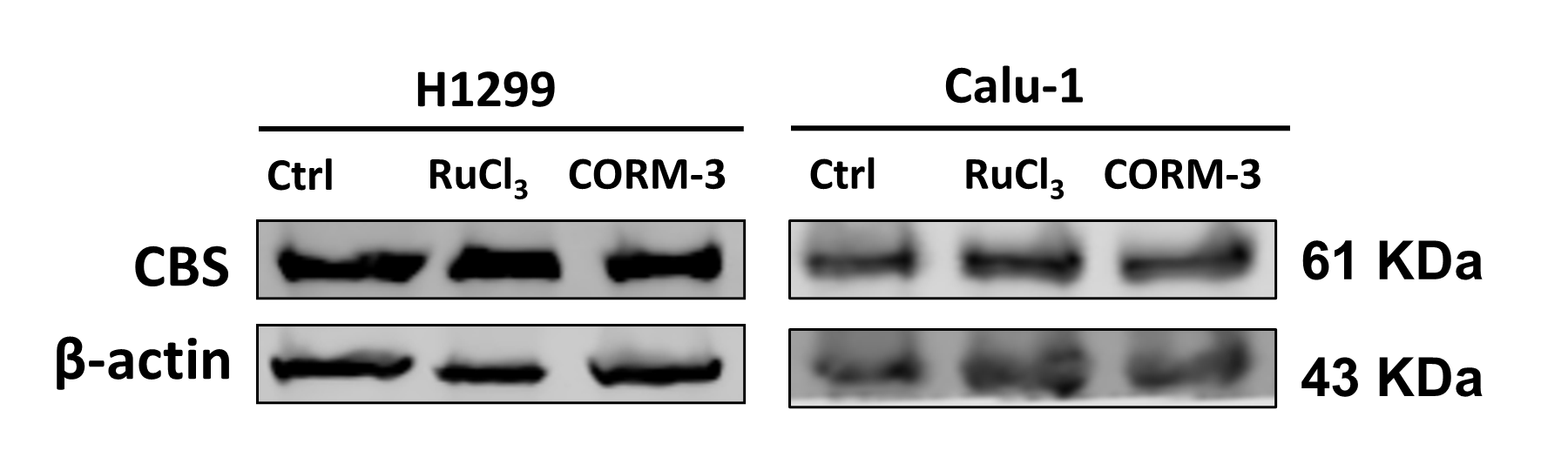

Supplement: Supplementary file 2 — Extended Data 1 [file 41420_2023_1743_MOESM2_ESM.tif]
